# Determinants for Simultaneous Binding of Copper and Platinum to Human Chaperone Atox1: *Hitchhiking not Hijacking*

**DOI:** 10.1371/journal.pone.0070473

**Published:** 2013-07-30

**Authors:** Maria E. Palm-Espling, C. David Andersson, Erik Björn, Anna Linusson, Pernilla Wittung-Stafshede

**Affiliations:** Department of Chemistry, Umeå University, Umeå, Sweden; Russian Academy of Sciences, Institute for Biological Instrumentation, Russian Federation

## Abstract

Cisplatin (CisPt) is an anticancer agent that has been used for decades to treat a variety of cancers. CisPt treatment causes many side effects due to interactions with proteins that detoxify the drug before reaching the DNA. One key player in CisPt resistance is the cellular copper-transport system involving the uptake protein Ctr1, the cytoplasmic chaperone Atox1 and the secretory path ATP7A/B proteins. CisPt has been shown to bind to ATP7B, resulting in vesicle sequestering of the drug. In addition, we and others showed that the apo-form of Atox1 could interact with CisPt *in vitro* and *in vivo*. Since the function of Atox1 is to transport copper (Cu) ions, it is important to assess how CisPt binding depends on Cu-loading of Atox1. Surprisingly, we recently found that CisPt interacted with Cu-loaded Atox1 *in vitro* at a position near the Cu site such that unique spectroscopic features appeared. Here, we identify the binding site for CisPt in the Cu-loaded form of Atox1 using strategic variants and a combination of spectroscopic and chromatographic methods. We directly prove that both metals can bind simultaneously and that the unique spectroscopic signals originate from an Atox1 monomer species. Both Cys in the Cu-site (Cys12, Cys15) are needed to form the di-metal complex, but not Cys41. Removing Met10 in the conserved metal-binding motif makes the loop more floppy and, despite metal binding, there are no metal-metal electronic transitions. *In silico* geometry minimizations provide an energetically favorable model of a tentative ternary Cu-Pt-Atox1 complex. Finally, we demonstrate that Atox1 can deliver CisPt to the fourth metal binding domain 4 of ATP7B (WD4), indicative of a possible drug detoxification mechanism.

## Introduction

Cisplatin (cis-PtCl_2_(NH_3_)_2_; here abbreviated CisPt) is a commonly used anticancer agent for treatment of a variety of cancers, including; testicular, head, neck, bladder and lung [1], [2]. Its anticancer activity arises from forming stable adducts with DNA in the nucleus, thus interfering with replication and transcription. Initial results of CisPt treatment are often good but can decline over time due to development of resistance. The resistance can be either acquired or intrinsic and seems to be multifactorial [3]. One of the proposed mechanisms for CisPt resistance is involvement of the cells own copper (Cu) transporting system.

**Figure 1:**
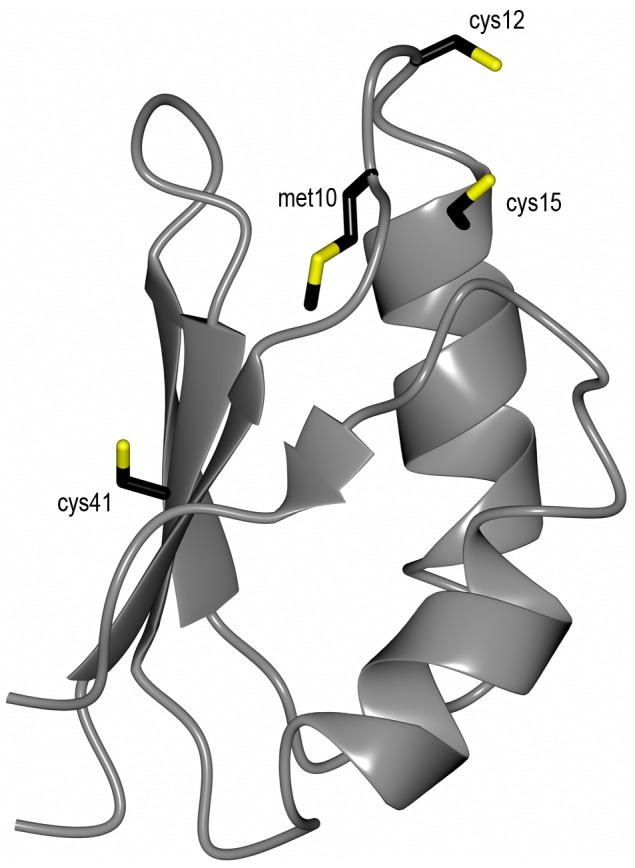
The copper chaperone Atox1. Cartoon model of Atox1 made in Ccp94 using PDB 1TL4 [16]. Stick residues are the two cysteines (Cys12 and Cys15) in the Cu-binding site, the 3:rd cysteine in the protein (Cys41) and methionine (Met10) in the conserved MxCxxC motif.

Cu transporter 1 (Ctr1), a membrane pump responsible for the cellular uptake of Cu, has been shown to play a major role in CisPt uptake and cytotoxicity [4]. The absence of the protein render cells resistant to CisPt [5] and patients with high levels of Ctr1 in their tumors responded better to platinum drug treatment [6]. The P_1B_ type Cu-transporting ATP:ases ATP7A and ATP7B are part of the secretory pathway and positioned in the trans-Golgi network. CisPt has been shown to trigger re-localization of these proteins towards more peripheral parts of the cell where they are thought to mediate CisPt resistance by sequestering the drug into intra-cellular vesicles [7], [8]. The ATP7A/B proteins have been found to be over-expressed in CisPt resistant carcinoma cells [9], [10]. ATP7A/B are multi-domain membrane spanning proteins with six, structurally similar, N-terminal metal-binding domains extending into the cytoplasm. The metal-binding domains are connected by peptide linkers and each domain has an α/β ferredoxin-like fold and a surface-exposed metal-binding motif: GMX**C**XX**C** [11], [12]. They can each bind one Cu via the Cys residues in this motif *in vitro*, although the role of Cu-binding to these domains *in vivo* may be regulatory [13]. The metal binding domains of ATP7B have been shown to interact with CisPt via their Cu sites [14], [15] and such interactions were found to be essential for ATP7B to mediate resistance [15]. The Cu chaperone Atox1 is the link between Ctr1 and ATP7A/B and transports Cu from Ctr1 to ATP7A/B in the cytoplasm. Atox1 has a similar structure and Cu-binding site as the ATP7A/B metal binding domains (**Figure 1**) [16].

There are a few studies that have reported CisPt binding to Atox1. Two crystalline forms of CisPt bound to Atox1 was reported and in both was Pt bound to the cysteines in the metal site. In one structure, CisPt was stripped of its original ligands and bound to an Atox1 monomer with additional coordination to a Tris-2-carboxyethyl-phosphine molecule (TCEP, the reducing agent used in the experiment). In the other structure, CisPt cross-linked two Atox1 molecules by binding to Cys15 from each metal binding site [17]. Using in-cell NMR, CisPt was shown to bind in the metal sites of Atox1 molecules that were over-expressed in *E. coli* cells [18]. Upon incubation Atox1 dimerized in the NMR tube, due to CisPt-promoted cross-links between proteins. Moreover, from an *in vivo* perspective, both *Drosophila* Atox1^−/−^ mutants [19] and mouse Atox1^−/−^ fibroblast cell lines [20] have been found to have lower sensitivity to CisPt than the wild types [19], [20]. Also, it has been shown that Atox1 is required for Ctr1-mediated uptake of CisPt into cells [20] and that down-regulation [19] or de-functionalization [20] of Atox1 contribute to CisPt resistance [19], [20].

In addition to the above studies, we recently showed that CisPt (and other Pt derivatives) can bind to both apo- and Cu-loaded forms of Atox1 *in vitro* [13], [21]. For the Cu-loaded form of Atox1, CisPt binding resulted in new spectroscopic signals that we proposed to arise due to Pt-Cu metal-metal interactions [13]. In small complexes, Pt(II) and Cu(I) are indeed known to form metal-metal bonds of the d^8^-d^10^ orbital overlap type [22], [23], [24], [25]. However, such metal-metal interactions have mostly been studied in small chemical complexes and are not well characterized in proteins [26]. We found in our original study that CisPt interaction with Atox1 promotes slow unfolding of the protein, followed by Pt-triggered aggregation [13]. This result suggested that Atox1 may act as a dead-end scavenger of CisPt *in vivo*, but transfer of CisPt from Atox1 to other proteins was not tested. Albeit that Atox1 is a Cu-transport protein, and *in vivo* it will likely spend a large fraction of its time in the Cu-loaded holo-form, there has been no Cu present in the reported studies of CisPt-Atox1 interactions [17], [18]. In addition to our work, there is only one other study that addressed the role of Cu: therein, it was suggested that Cu is expelled upon Pt binding to ATP7B’s metal-binding domain 6 although this was not proven [15].

Here we pin down the binding site for CisPt in the Cu-loaded form of Atox1 using strategic point-mutated variants and various biophysical/biochemical methods *in vitro*. We prove that both metals can bind simultaneously and that the unique spectroscopic signals originate from an Atox1 monomer species. Using quantum calculations, we define a tentative low-energy structural model of the ternary Cu-Pt-Atox1 complex. Finally, we show that mixing of Pt-bound Atox1 with the fourth metal binding domain of ATP7B (WD4) results in Pt transfer to WD4 and heterodimer formation.

## Materials and Methods

Cisplatin, dithiothreitol (DTT), CuCl_2_ and 2-(N-morpholino)ethanesulfonic acid (MES) were purchased from Sigma-Aldrich (Sweden). CisPt was dissolved in water, 2 mg/ml, and was shortly heated in the microwave to enhance solubility. The CisPt stock was incubated and prehydrolyzed for three days and used within five days to avoid degradation products. The protein sample buffer was 50 mM NaCl, 20 mM MES, pH = 6 and the reducing agent DTT. The DTT concentration in samples was kept fivefold higher than protein concentration. Copper was added to the samples as CuCl_2_ (20 mM in water) and immediately reduced to Cu^1^ by the DTT in the buffer.

### Protein Preparation

Expression and purification of WT Atox1 was published previously [13] and the method is based on the procedure of Kihlken, M.A et al [27]. The Atox1 WT plasmid was kindly provided by A. C. Rosenzweig (Northwestern University, Evanston, IL). Atox1 mutant Met10Ala (Met10 → Ala10) was reported previously [28] and plasmids for Atox1 mutants Cys41Ala (Cys41 → Ala41) and 3Cys3Ala (Cys12, Cys15, Cys41 → Ala12, Ala15, Ala41) were ordered from Gene Script Corporation (Piscataway, NJ) in pET-21b(+) vectors. Plasmids were sequenced to confirm mutations and proteins were expressed and purified like WT Atox1. Protein masses were confirmed with mass spectrometry. The Atox1 mutant Cys15Ala (Cys15 → Ala15) was kindly provided by Moritz S. Niemiec (Umeå University, Umeå, Sweden) and WD4 was prepared as before [29]. ε_280_ = 1490 M^−1^ cm^−1^ for WD4 and ε_280_ = 2980 M^-1^ cm^-1^ for Atox1 was calculated based on sequence.

### CD Spectroscopy

Circular dichroism (CD) spectra were recorded on a Jasco J-720 Spectropolarimeter at 20°C. Near-UV CD (260–400 nm) was measured with protein concentration of 50 µM in a 1 cm cell. Samples were incubated 10 min before measurement. 0–5 eq. CisPt was titrated to holo- (pre-incubated with 1 eq. CuCl_2_) protein in one set of titrations and 0–5 eq. CuCl_2_ was added to premixed 1∶1 protein-CisPt in another set. Cu-binding was separately assured with titration of CuCl_2_ to apo-proteins (data not showed). Baselines with buffer were subtracted from each CD-spectrum. Time dependences of CisPt induced protein unfolding were monitored by far-UV CD (200–320 nm) and was analyzed at 220 nm. 50 µM apo-protein and added 1 eq. CuCl_2_ and/or 5 eq. CisPt in sealed cyvettes at 20°C were followed over the time for two weeks. CD at 220 nm as a function of time were fitted to single exponential decays (Kaleidagraph) to obtain apparent rate constants for CisPt induced unfolding. t_1/2_ was calculated as ln2/*k* where k is the first order rate constant.

### Analytical Gelfiltration SEC (Size Exclusion Chromatography)

Analytical gelfiltration was performed with a Superdex 75 10/300 analytical column (GE Healthcare, column volume = 24 ml) on an ÄKTA purifier (GE Healthcare) at 6°C. Running buffer used was 40 mM TrisHCl, 50 mM NaCl, pH = 7.6. Protein concentration was 150 µM and injection volume 100 µl. For holo-samples 1 eq. CuCl_2_ was pre-added, and to apo- and holo- samples 1 or 5 eq. CisPt was added. Samples were incubated for 10 minutes before SEC-analysis. Dual channel detection at 280 nm (protein) and 254 nm (for monitoring sulfur-metal bonds) was employed.

For CisPt transfer experiments 50 µM Atox1 or WD4 was mixed with 2 eq. CisPt and incubated for 2 h at 20°C. Analytical gelfiltration was run as described above and the protein monomer peak (14.2 ml for Atox1 and 12.8 ml for WD4) was collected and concentrated. In this way free CisPt and potential dimmers were removed. Next, 0.5 eq. of the second protein (Atox1 or WD4) was added and the sample was incubated for 4 h in RT. After analytical gelfiltration the protein peaks were collected individually and Pt-content was measured with ICP-MS. Protein concentration was estimated by calculating area under curve for collected fractions.

To allow near-UV CD analysis of peaks from analytical gelfiltration, a 500 µM Atox1 sample with 1 eq. CuCl_2_ and 3 eq. CisPt was prepared. The sample was incubated for 10 minutes and analytical gelfiltration was performed as described above. The protein peaks for monomeric and dimeric Atox1 were collected and immediately analyzed by near-UV CD as described above. The measurement were repeated with the order of measuring CD on the two peaks reversed to assure that any time delay did not affect the result. Far-UV CD of the protein samples to control that protein was folded was performed as above in a 0.1 cm cell.

### SDS-gel Analysis

SDS-PAGE (Sodium dodecyl sulfate - polyacrylamide gel electrophoresis) was used for analysis of CisPt induced protein oligomerization and aggregation. Protein samples (50 µM) with apo-protein and addition of 1 eq. CuCl_2_ and/or 5 eq. CisPt were mixed and incubated at 20°C for various times (4 days, 2 days, 1 day, 4 h and freshly made). SDS-dye was added to the samples and samples were loaded onto a 16% SDS-gel under non-reducing conditions (no boiling).

### Assessing Possible Loss of Cu upon Pt-binding

Atox1 samples (50 µM) were prepared with 1 eq. CuCl_2_ and incubated for 10 minutes. The samples were diluted three times with buffer and concentrated to get rid of unbound Cu. 1 eq. CisPt was added to the samples and the samples were incubated for various times; 20 min, 40 min, 2 h and 6 h. The samples were diluted three times with buffer and concentrated. The flow through was collected and Cu-content measured with ICP-MS. The samples were replicated three times and all samples compared with controls were CisPt additions were excluded. Additional control experiments were made in the absence of Atox1 but with the same Cu (and CisPt) concentrations as above.

### ICP-MS

Samples for ICP-MS analysis were diluted ten times with water and a final concentration of 10% of aqua regia (1∶3 HNO_3_:HCl) to a final volume of 1300 µl. A PerkinElmer/Sciex Elan DRC-e ICPMS instrument was used for the measurements. Quantification was done by matrix matched standards and the isotopes ^63^Cu^+^, ^65^Cu^+^, ^194^Pt^+^ and ^195^Pt^+^ were monitored to verify the absence of spectral interferences.

### Quantum Mechanical Calculations of Di-metal Site

Calculations were performed on the NMR-derived structure of Atox1, 1TL4 [16], using three different Atox1 conformations (A, B and C), representative of the NMR structured ensemble. CisPt^+^ (Cl(NH_3_)_2_Pt^+^) was built into the structures in several theoretically plausible binding modes to the Cys12 sulfur. Cys12 is the more accessible binding partner for CisPt^+^ of the two cysteines binding Cu. The protein structures were truncated to include amino acids in the proximity of the Cu-binding site including Thr11, Cys12, Gly13, Gly14, Cys15, and Lys60 (side chain N (A and C) or NCH_3_ (B)). The Atox1-CisPt^+^ complexes were geometry optimized with Turbomole [30] in two steps using dispersion-corrected density functional theory (DFT-D3) [31] in gas phase with the b-lyp functional [32], [33], [34], a multiple grid size “m3”, basis set def2-SVP [34] (step1) and def2-TZVP [34] (step2), and an effective-core potential (ECP) def2-ecp for Pt and the Stuttgart-Koeln MCDHF RS [35] for Cu. CisPt^+^, Cu and Cys12 and Cys15 sulfurs were allowed to move during optimization, all other atoms were fixated. Single point energies were calculated using the same DFT settings as in geometry optimization step2 for the optimized structures of the Atox1-CisPt^+^ complex, and both species separately. Interaction energies, ΔE, were computed by subtracting the summarized energy for CisPt^+^ and Atox1 from the Atox1-CisPt^+^ complex energy. More details regarding the computational setup is given in the **File S1**.

## Results and Discussion

### Open Questions and Protein Engineering Approach

To define the binding site for CisPt in the Cu-loaded form of Atox1, we selected four strategic Atox1 variants for our work: (1) 3Cys3Ala Atox1 in which Atox1’s three Cys are exchanged for Ala, (2) Cys41Ala Atox1 in which the Cys not involved in the Cu site is exchanged for Ala, (3) Met10Ala Atox1 in which the Met in the conserved Cu-binding loop (**M**XCXXC) is exchanged for Ala, and (4) Cys15Ala in which the second of the Cu-binding Cys (MXCXX**C**) is exchanged for Ala.

Based on near-UV CD in the 300–350 nm range and unique NMR chemical shifts appearing only for the mixture containing both metals, we previously proposed that Pt binds to Cu-Atox1 without expulsion of Cu [13]. With the above variants and a biophysical experimental approach, here we set out to explicitly identify the binding site(s) for Pt in the Cu-loaded form of Atox1 and test if the di-metal site is formed in a monomeric or higher order species of the protein. Since Atox1 was found as a dimer in the crystal structure [17] one may speculate on a bridging metal cluster. Moreover, it was recently shown that MoO_4_ forms a metal-cluster with Cu-Atx1 (yeast analog) involving a trimer [36]. Finally, to find clues for the drug detoxification mechanism *in vivo*, we test if CisPt can be transferred from Atox1 to the fourth metal-binding domain of ATP7B (WD4, which is the next protein in the Cu-transfer chain) prior to Pt-induced Atox1 unfolding.

### Cu-Pt Interactions in Atox1 Variants

**Figure 2:**
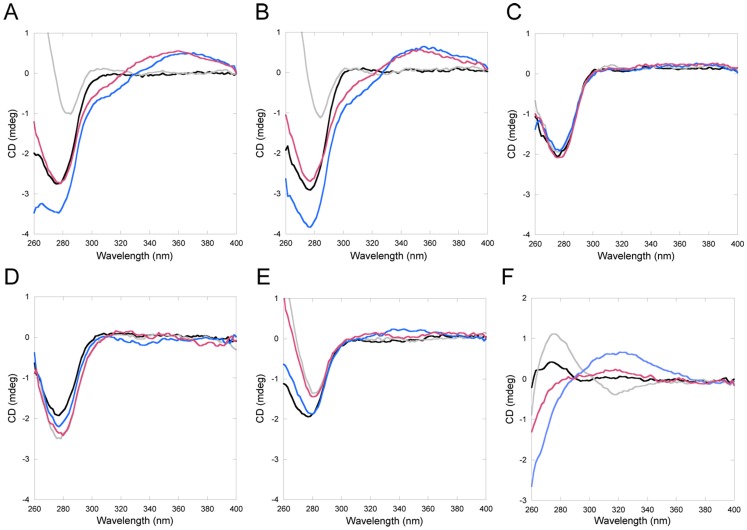
Near-UV CD for Atox1 variants and WD4. Selected traces from Cu/CisPt titrations. Black: Apo-protein. Grey: +1 eq. Cu. Blue: +1 eq. Cu and +2 eq. CisPt. Red: +1 eq. CisPt +2 eq. Cu. **A**. WT Atox1. **B**. Cys41Ala Atox1. **C**. 3Cys3Ala Atox1. **D**. Cys15Ala Atox1. **E**. Met10Ala Atox1. **F**. WT WD4. See also **Figure S1**.

All Atox1 mutants can bind Cu stoichiometrically *in vitro*, except 3Cys3Ala Atox1. For Cys15Ala Atox1, Cu binding only occurs in combination with dimer formation (unpublished data and [37]). Cu binding to Atox1 can be detected through changes in the near-UV CD spectrum around 260 nm, due to Cu-Cys bond contributions [38]. Addition of CisPt to wild-type (WT) Cu-Atox1 (or Cu addition to Pt-bound WT Atox1) results in new CD signals around 300–350 nm. These low energy bands (above 300 nm) are indicative of metal-metal interactions, since aromatic residue contributions to the near-UV CD (Atox1 has one Phe and two Tyr) will appear below 300 nm [39]. We note that we cannot exclude that there is a bridging atom between the two metals, for example a solute molecule. In **Figure 2**, we show the CD signals for the variants upon the addition of Cu and CisPt (see also **Figure S1**). It is clear that only for wild-type and Cys41Ala Atox1 variants, are new CD features above 300 nm found. Cys3Ala3 Atox1 does not bind Cu and thus, this experiment cannot conclude if CisPt is bound or not to this variant as CisPt binding itself does not change the CD spectrum. Other experiments show that both Met10Ala and Cys15Ala Atox1 variants also bind CisPt (see below), but the presence of both metals apparently does not result in a complex that govern spectroscopically-detected metal-metal interactions. These results imply that a complete MXCXXC motif is needed in Atox1 to provide a foundation for electronic overlap between Pt and Cu.

### Pt-triggered Atox1 Unfolding

**Figure 3:**
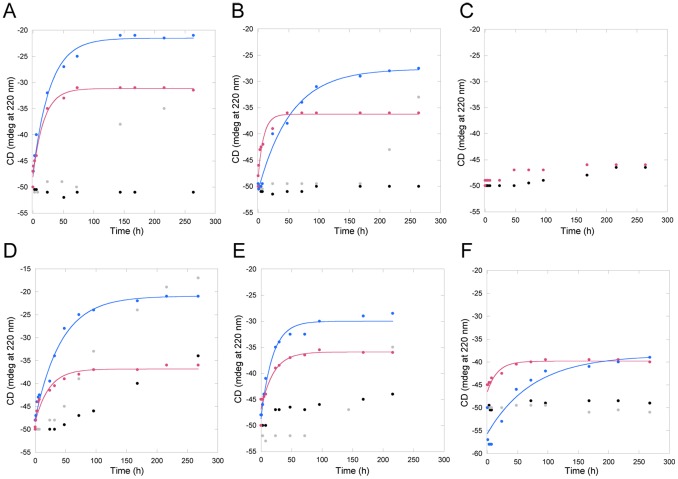
CisPt-triggered unfolding of Atox1 variants and WD4. Black: Apo-protein. Grey: +1 eq. Cu. Blue: +1 eq. Cu and +5 eq. CisPt. Red: +5 eq. CisPt. **A**. WT Atox1. **B**. Cys41Ala Atox1. **C**. 3Cys3Ala Atox1. **D**. Cys15Ala Atox1. **E**. Met10Ala Atox1. **F**. WT WD4.

As a hallmark of CisPt binding to Atox1 is Pt-induced protein unfolding with speed depending on ratio of CisPt to protein and total concentrations [13]. For protein and drug concentrations in the micromolar range, as used here, CisPt-triggered unfolding of Atox1 at 20°C occurs over 1–2 days with a half-life around 10–30 hours. We compared CisPt-induced unfolding for apo- and holo-forms of all Atox1 variants (no holo-form for 3Cys3Ala). The resulting data at 220 nm as a function of time is shown in **Figure 3**. The major differences between apo- and holo-forms are that extent of Atox1 unfolding is larger for holo-forms, whereas CisPt-triggered unfolding of apo-forms proceeds somewhat faster. Tentative explanations to this are that the presence of Cu helps promote unfolding and aggregation at later stages in the reaction (perhaps because of instability of Cu-Pt cluster), and that since the apo-forms are less thermally stable than holo-forms [38], initial lower protein stability facilitates faster destructive consequences of CisPt binding. Notably, the holo-form of Met10Ala Atox1 unfolds faster than holo-forms of WT and Cys41Ala Atox1 variants upon CisPt addition (**Table 1**). This is in agreement with low protein stability for the holo-form of the Met10Ala variant [40].

**Table 1:** t_1/2_ of CisPt induced unfolding of apo- and holo-Atox1 variants and WD4 WT.

	t_1/2_ apo (h)	t_1/2_ holo (h)
**Atox1 WT**	12.3±0.2	19.4±2.3
**Atox1 cys41ala**	6.3±1.1	37.2±3.9
**Atox1 3cys3ala**	No unf.	
**Atox1 cys15ala**	15.7±2.7	29.6±3.2
**Atox1 met10ala**	14.2±2.9	13±1.5
**WD4 WT**	11.5±3.9	52.4±17.9

5 eq. CisPt was added to either apo- or holo- (+1 eq. Cu) protein and loss of secondary structure was measured by far-UV CD in RT over two weeks. CD data at 220 nm plotted vs. time and fitted to single exponential decays.

3Cys3Ala Atox1 does not show any unfolding during the course of the experiment, demonstrating that when all three Cys are exchanged to alanines, there is no CisPt binding. This result also shows that the effects we observe with the Met10Ala Atox1 mutant are indirect and not due to the elimination of Met10 as a binding site for CisPt. Cys41Ala Atox1 exhibits similar unfolding profiles as the wild-type protein. Thus the third peripheral Cys41 is not involved in CisPt-induced Atox1 unfolding, but instead the Pt binding near the Cu is responsible for promotion of protein unfolding. The Cys15Ala variant is difficult to analyze as the holo-form of the protein dimer is unstable and starts to unfold by itself upon incubation. Nonetheless, the presence of CisPt increases the speed of Cys15Ala Atox1 unfolding, indicative of CisPt binding to this variant.

**Figure 4:**
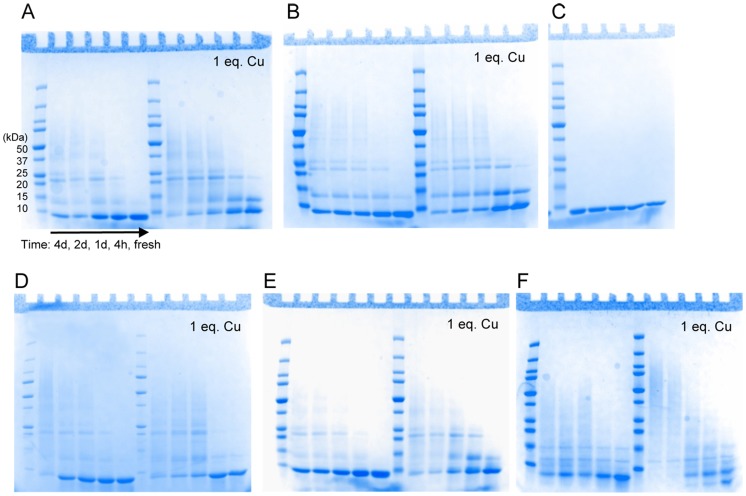
SDS-gel analysis of CisPt induced Atox1 aggregation. Apo- and +1 eq. Cu samples treated with 5 eq. CisPt over the time of 4d, 2d, 1d, 4h and fresh made. **A**. WT Atox1. **B**. Cys41Ala Atox1. **C**. 3Cys3Ala Atox1. **D**. Cys15Ala Atox1. **E**. Met10Ala Atox1. **F**. WT WD4. See also **Figure S2**.

SDS gels show the appearance of aggregation products of Atox1 variants mixed with CisPt as a function of incubation time (**Figure 4**). In contrast, incubation of apo- and holo-forms of all Atox1 variants does not result in any protein aggregation during the same time-span (**Figure S2**). The Cys3Ala Atox1 variant, as expected, does not show any aggregation in the presence of CisPt, again supporting that no CisPt becomes bound. Taken together, the unfolding and aggregation experiments support that CisPt binds to the Cu-forms of WT, Cys41Ala, Met10Ala, and Cys15Ala Atox1 variants. We earlier identified the presence of both Cu and Pt in unfolded aggregates of WT Atox1 [13].

### Biochemical Evidence for No Loss of Cu upon CisPt Binding to Cu-Atox1

**Figure 5:**
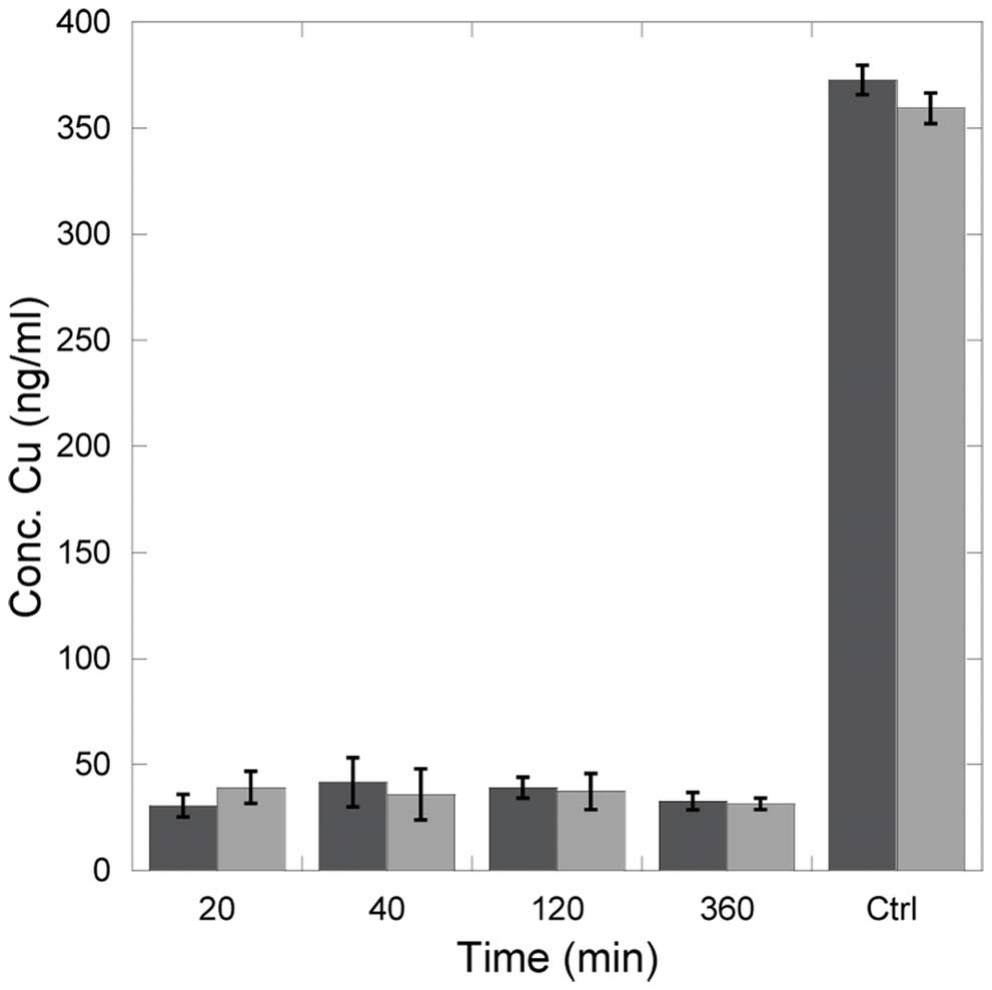
No loss of Cu upon CisPt binding to Atox1 WT. Samples treated with 1∶1 of CisPt and incubated for various times. Samples were centrifuged and Cu concentration was measured in flow trough (cut off in filter 3000 Da). Dark grey: CisPt treated holo Atox1. Light gray: Holo-Atox1. The last two columns are positive controls where Atox1 was omitted.

Although spectroscopic data strongly indicate that both metals are bound at the same time to Atox1, we wanted to test this explicitly using a direct physical method. Therefore, Cu-Atox1 was mixed with CisPt and such samples were incubated for different lengths (0–6 h) followed by filtration (cut off 3000 Da) and determination of Cu content of flow through (by ICP-MS). A control experiment with a solution of only Cu (no protein) reveals the presence of about 350 ng/ml of Cu. Thus, this is the Cu-level we would expect if Cu is expelled upon CisPt binding. Experiments with Cu-Atox1 using the same procedure but no addition of CisPt demonstrate little loss of Cu. We find that in all samples of Cu-Atox1 mixed with CisPt, less than 10% of all Cu in the sample is present in the flow through (**Figure 5**), and this level is similar to the Cu-Atox1 samples without CisPt. Probably there is some leakage of holo-protein through the filter contributing to the detected 10% Cu. Thus, we conclude that despite of CisPt binding, the Cu in Atox1 remains bound.

### Oligomeric Status of Pt-Cu-Atox1 Complexes

**Figure 6:**
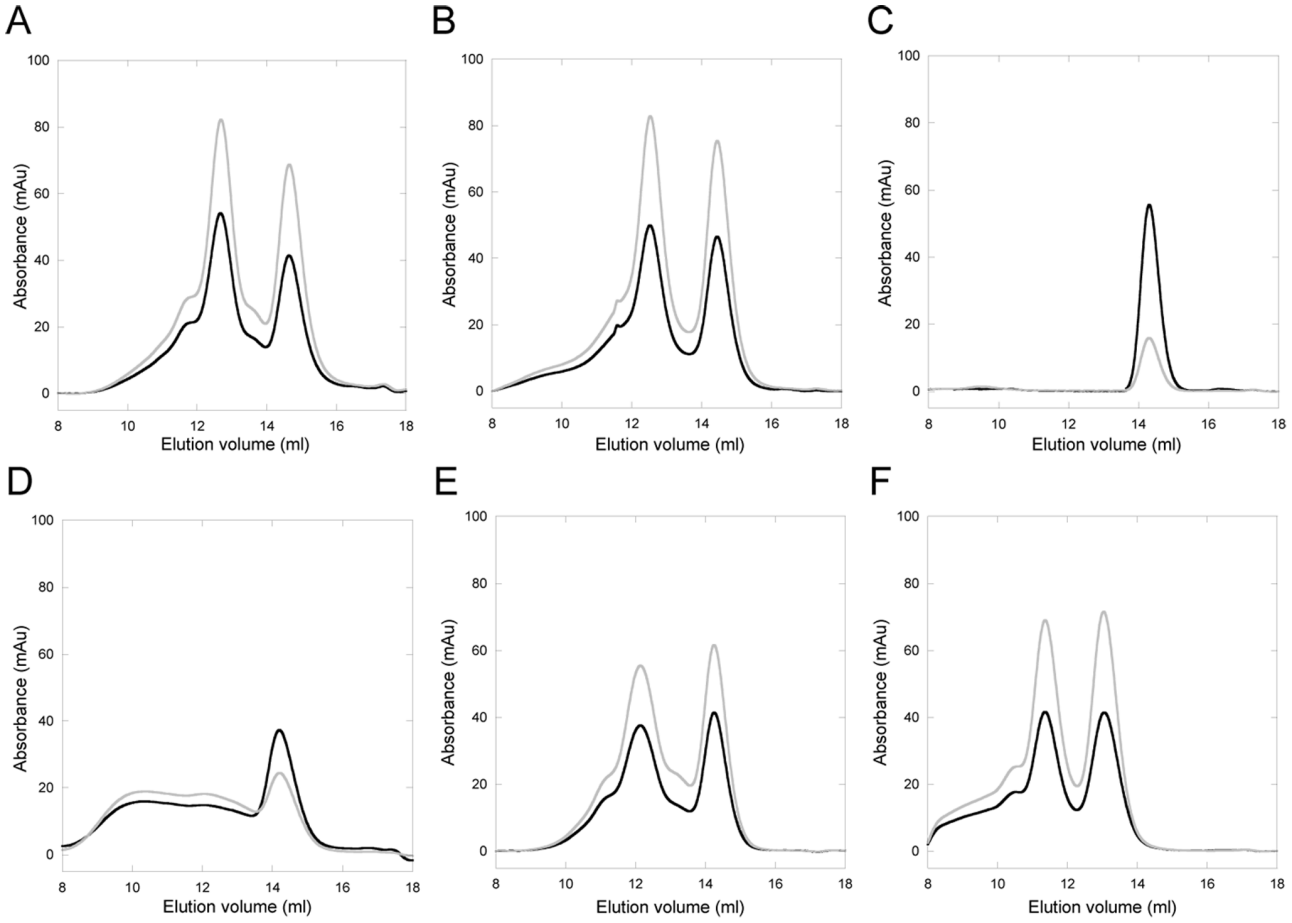
Analytical gelfiltration of Atox1/WD4. Protein +1 eq. Cu and 5 eq. CisPt. Black: 280 nm. Grey: 254 nm. **A**. WT Atox1. **B**. Cys41Ala Atox1. **C**. 3Cys3Ala Atox1. **D**. Cys15Ala Atox1. **E**. Met10Ala Atox1. **F**. WT WD4. Sample incubation time is 10 min. See also **Figure S3–S8**.

Size-exclusion chromatography can be used to assess Cu-loading (via 280/254 nm absorption ratio) as well as complex formation (via elution position). We earlier used this method to characterize Cu transfer from Atox1 to WD4 of ATP7B and hetero-complex (Atox1-Cu-WD4) formation [29]. Apo- and Cu-forms of Atox1 (and all variants except Cys15Ala) are monomeric when analyzed by SEC (**Figures S3-S7**). The elution profile for Cys15Ala Atox1 reveals that in the presence of Cu, oligomers form (prominent dimer peak and a wide range of larger oligomers) and these, but not the monomer band, contain Cu. When WT Atox1 is mixed with 1 eq. of CisPt, it remains a monomer whereas treatment with excess CisPt results in monomers mixed with higher oligomeric species. In contrast, when Cu-Atox1 is treated with 1 or 5 equivalents of CisPt or preformed CisPt-Atox1 (1∶1) is treated with Cu, the elution pattern contains two distinct peaks: monomers and dimers. Based on the 254/280 ratio, both peaks contain metal (**Figure 6**). Atox1 dimers were found in the crystal structure of Cu-Atox1 [41] and in one of the crystal forms of Pt-Atox1 [17]. In both cases, the proteins interact with the metal via the metal-site cysteines in the dimer.

Similar monomer/dimer patterns are observed for Cys41Ala Atox1, emphasizing the lack of role for Cys41 in Pt binding, and for Met10Ala Atox1. The fact that Met10Ala Atox1 behaves similarly to WT Atox1 in SEC (and in CisPt-triggered unfolding/aggregation reactions) indicates that CisPt binds to the Cu-form of Met10Ala Atox1 in the same way as CisPt binds to WT Cu-Atox1. We speculate that it is the increased loop dynamics, due to the absence of Met10 [40], which limits Pt-Cu electronic overlap.

In agreement with the unfolding data, 3Cys3Ala Atox1 remains monomeric during all additions of CisPt and ICP-MS analysis show no protein bound Pt. For Cys15Ala Atox1, we observe monomers upon CisPt addition that according to ICP-MS analysis contains Pt, suggesting that Pt can bind to the sole Cys in the metal-binding site of the monomer. In the presence of both Cu and CisPt, there are Cys15Ala Atox1 monomers detected but also a range of oligomeric species. In contrast to WT, Met10Ala, and Cys41Ala Atox1 mixtures, a distinct dimer peak is not observed for Cys15Ala Atox1 in presence of both Cu and Pt. We speculate that this is due to fast unfolding of the Cu-form of this mutant (dimer) also in the absence of CisPt, thus promoting unfolded state oligomerization faster than in the case of the other Atox1 variants.

ICP-MS metal analysis of monomer and dimer fractions for WT and Met10Ala Atox1 revealed the presence of both metals in both fractions, in roughly equal amounts. For Cys15Ala Atox1, ICP-MS analysis showed that the monomer fraction contains little or no Cu (as expected) but more Pt, whereas the dimer/aggregate fraction contained both metals. Thus, Pt binding to Cu-Atox1 occurs in the monomer species and this triggers formation of dimers containing both metals. We note that the ICP-MS measurements are only qualitative. Quantitative analysis is hampered by partial precipitation of protein fractions when mixing with aqua regia.

### Monomer or Dimer Responsible for Unique CD Feature?

**Figure 7:**
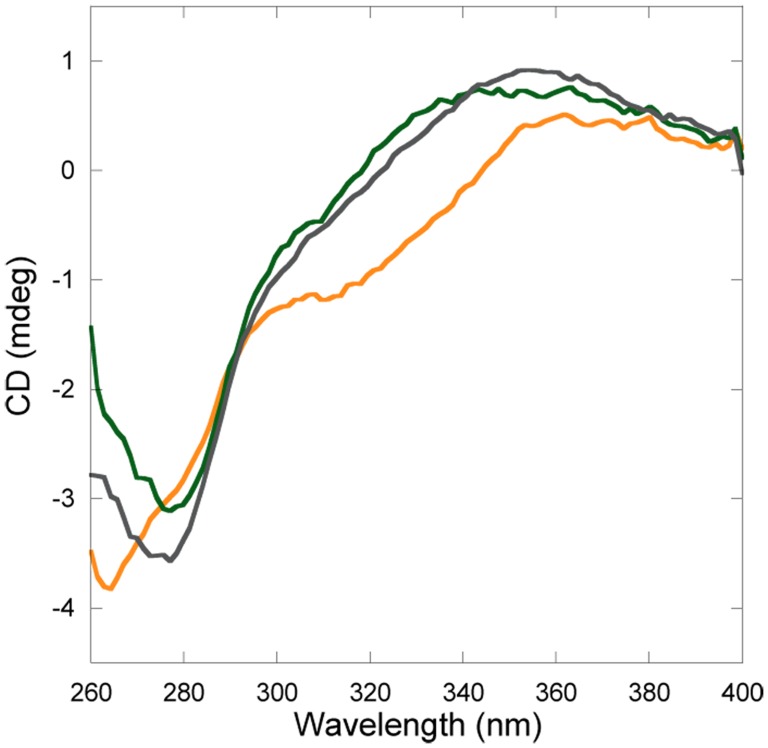
Near-UV CD of peaks from analytical gelfiltration. Atox1+1 eq. Cu +3 eq. CisPt. Green: Monomeric peak (14.2 ml). Yellow: Dimeric peak (12.8 ml). Grey: Atox1+1 eq. Cu +2 eq. CisPt. Data is normalized to correspond to 50 µM protein in all three cases.

Since Cu and Pt are found in both the monomeric and dimeric forms of Atox1, we wanted to determine which one of these species is responsible for the unique CD signals. Therefore, we performed the same SEC experiment as above but with higher protein/metal concentrations. After SEC analysis the monomer and dimer Atox1-Cu-Pt peaks were collected separately and immediately analyzed by CD. The resulting spectra (**Figure 7**) reveal that it is the monomer Atox1 fraction that gives rise to the distinct CD feature that dominates in spectrum in the non-purified mixtures. Still, the Atox1-Cu-Pt dimer exhibits CD absorption above 300 nm: we speculate that this is due to a metal-cluster formed when the two proteins interact via their metal sites.

### Modeling of Di-metal Site in Atox1

**Figure 8:**
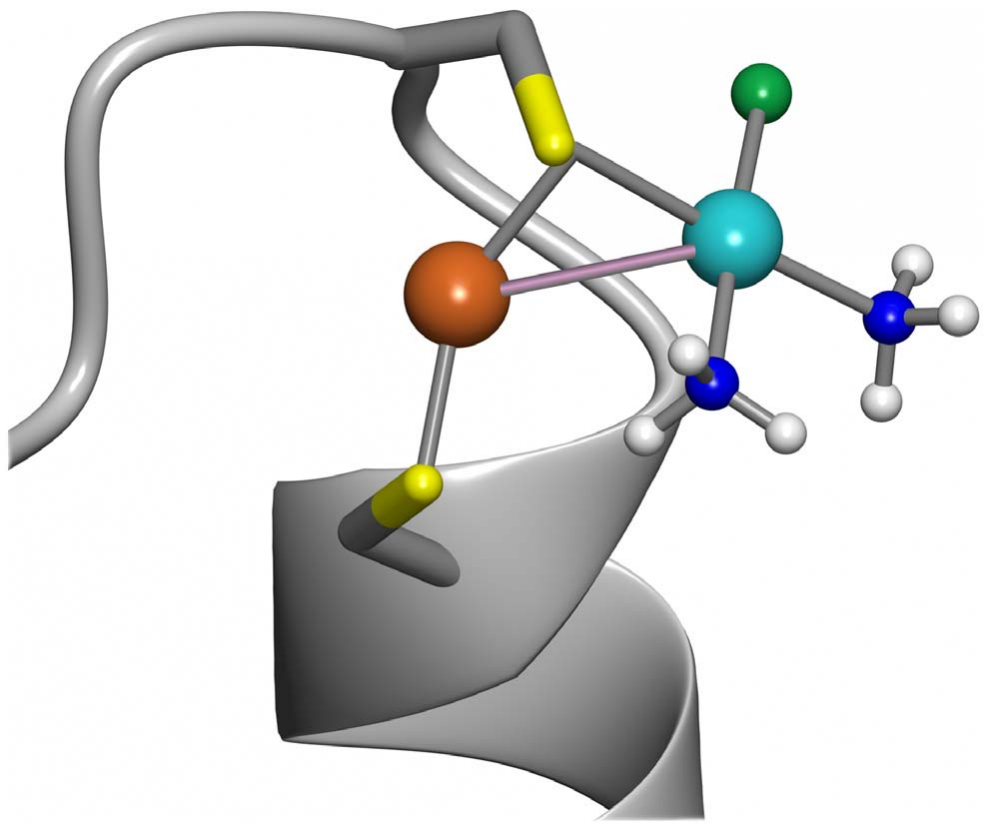
Geometry optimized model of Cu-loaded Atox1 with CisPt. Low-energy complex (A2) between Cu-Atox1 and CisPt^+^ calculated using DFT-D3. CisPt was positioned next to Cys12 in the holo-form, since this is the more accessible of the two cysteines and thus the most likely interaction site for Pt. Pt-Cu metal-metal interaction bond is shown in light purple. See also **supporting information**.

Low-energy binding modes for CisPt^+^ to the holo-form of WT Atox1 were computed by geometry optimization of Atox1-CisPt complexes using DFT calculations. The initial binding modes were generated by placing the CisPt^+^ at a probable binding location near the sulfur of Cys12. We selected Cys12 since it is more exposed to solvent than Cys15 in the folded structure, and, in fact, steric hindrance did not allow placement of CisPt adjacent to Cys15. Three protein conformations (A, B and C; **Figure S10**) and several CisPt starting geometries (A1–4, B1–2, and C1–4) were optimized. The optimized structures A2 and A3 had the strongest interaction energies (ΔE, Table S1 in **File S1**) between Cu-Atox1 and CisPt^+^ and the A2 complex is shown in **Figure 8**. In these complexes, the average Pt-S distance and Pt-Cu distance was 3.37 Å and 3.39 Å, respectively. The average Pt-Cu distance was 3.41 Å when including all low-energy complexes (A2, A3, B1 and C4; **Figure S11**) for all three protein structures. The Pt-Cu distance found in Atox1 is longer than previously reported values for Pt(II)-Cu(I) dative bonds (2.5–3.0 Å [22], [23], [42], [43], [44]) although we note that transition metal-metal bonds may be up to 3.6 Å [45], [46].

The use of a static protein structure may have hindered CisPt from achieving a more optimal binding mode to Cu-Atox1 that may have been accessible if protein dynamics was allowed. Taken together, the geometry optimizations show that there are several low energy complexes where CisPt^+^ binds to the sulfur of Cys12. The distance between Pt and Cu is small enough for the two atoms to form a metal-metal interaction in a flexible system.

### Similar CisPt Reaction with ATP7B’s WD4?

To study possible transfer of CisPt from Atox1 to the next protein in the Cu-transport chain, ATP7B, we selected the 4^th^ metal-binding domain of ATP7B (WD4). We have earlier characterized the transfer of Cu between these two proteins using SEC and ITC [29]. An important property of WD4 that makes the experiments possible is that, despite the same size as Atox1, it elutes as an apparent higher molecular weight species and the two proteins are thus separated upon SEC.

First we tested if WD4 could bind CisPt when the metal was added to the solution. In other studies, WD1–4 was shown to interact with three Pt ions, suggesting that at least 3 of the 4 domains can bind Pt [14]. Moreover, WD6 has been shown to bind Pt in a reaction that leads to slow protein oligomerization [15]. Based on the similarity to Atox1, we expect WD4 to bind CisPt like Atox1 and that many of the biophysical characteristics determined for Atox1-Pt will hold true also for WD4-Pt. **Figure 2F** demonstrates that Cu-WD4 can bind CisPt resulting in similar unique CD features as for Atox1 and indicative of Pt-Cu electronic overlap. From **Figure 3F** it is noted that CisPt-induced protein unfolding is reduced for WD4: the kinetics is slower and the extent of unfolding is less for WD4 compared to for WT Atox1 (especially in the holo-forms). Nonetheless, gel analysis reveals protein aggregation patterns similar, or even more exaggerated, for WD4 as compared to for Atox1 (**Figure 4F**). SEC analysis of WD4 mixtures with Cu and CisPt reveal monomer and dimer peaks like the Atox1 variants (**Figure 6F** **,** **Figure S8)** and both monomer and dimer WD4 peaks contain both metals according to ICP-MS**.** Thus, WD4 can bind Pt and the ability of Pt transfer from Atox1 or vice versa can be tested.

### Transfer of Pt between Atox1 and WD4

**Figure 9:**
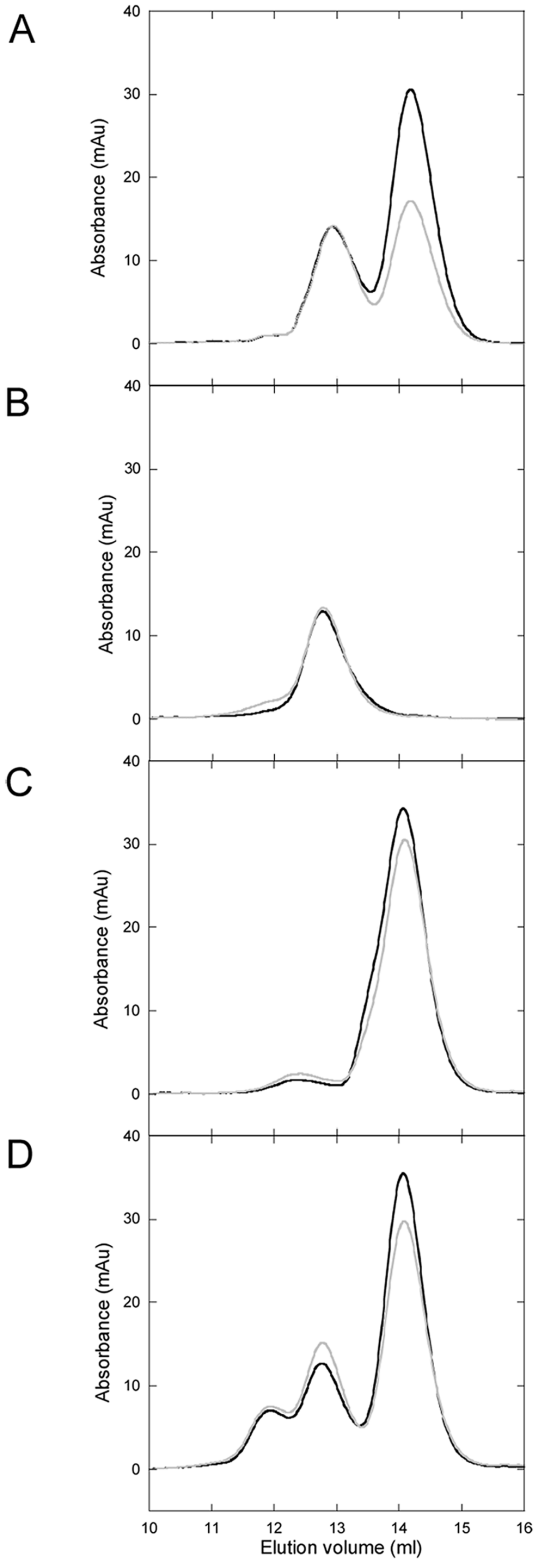
Pt-transfer from Atox1 to WD4. Analytical gelfiltration, Black: 280 nm, Grey: 254 nm. **A**. Apo-Atox1 mixed with 0.5 eq. apo-WD4. **B**. Control, WD4. No Atox1-CisPt added, otherwise experiment conducted as D. **C**. Control, Atox1-CisPt. No WD4 added, otherwise experiment conducted as D. **D**. Transfer experiment. Atox1-CisPt, filtrated and concentrated, mixed with 0.5 eq. WD4 and incubated for 4 h prior to SEC analysis. See also **Figure S9**.

Using SEC, we tested the ability of the monomeric Atox1-CisPt complex to deliver CisPt to WD4. First, the monomer fraction of a CisPt-Atox1 mixture was isolated (thus no free CisPt or oligomeric species present) and this was incubated with apo-WD4 before SEC analysis of products. Upon identical treatments, WD4 alone and CisPt-Atox1 alone samples result in single monomer bands. In contrast, when WD4 and CisPt-Atox1 is mixed, three peaks are detected (**Figure 9**). In addition to monomeric Atox1 and WD4 peaks, a third peak appears corresponding to a higher MW that suggests formation of a Pt-bridged Atox1-WD4 dimer. The elution profile is similar to that found in Cu-transfer experiments between Atox1 and WD4 [29]. Based on the 254/280 nm ratio and ICP-MS measurements on fractions, all three protein species contain Pt, in line with a Pt-dependent hetero-dimer and CisPt transfer to monomeric WD4. Notably, there appears to be an inverse correlation between pre-incubation time of Atox+CisPt *versus* degree of Pt transfer to WD4: longer pre-incubation of Atox1-Pt results in less Pt transfer to WD4 (data not shown). This trend can be linked to changes in Pt coordination with time: binding of Pt to the empty Cu-site in Atox1 likely starts with one Cys side chain, followed by loss of Pt ligands and interactions also with the second Cu-site Cys side chain. Therefore, the longer the incubation time, the tighter is Pt-binding to the apo-protein, and transfer to another protein will be less favored.

In **Figure S9**, we compare the SEC traces for the (Atox1-CisPt+WD4) transfer experiment with traces for CisPt-induced Atox1 dimerization and CisPt-induced WD4 dimerization. Notably, the dimer in the transfer experiment elutes in between the positions of two homodimers, in accord with a heterodimer.

### Conclusions

CisPt is the leading drug for difficult cancers despite its many side effects, involving protein interactions such as the Cu transporting proteins. Since a large portion of the supplied CisPt is believed to never reach DNA and kill cells, but remain in the cytosol [47] [48], there may be a huge gain from understanding (and eventually abolishing) unwanted CisPt-protein interactions. CisPt favors interactions with Met [49], [50] and Cys side chains, but other binding modes have also been noted [51], [52]. The Cu chaperone Atox1 has a conserved MXCXXC motif in which Cu binds to the two Cys residues. Here we show that Atox1 can bind Cu and CisPt simultaneously via the Cys residues in the CXXC motif allowing for the formation of metal-metal direct or bridged interactions. This results in d^8^-d^10^ electronic transitions that are visible in the CD spectrum above 300 nm. If the metal-binding loop in Atox1 is disturbed (via Met-to-Ala substitution at position 10), the metals can still bind but the electronic transitions do not take place, probably due to more dynamics in the metal-binding loop region. The di-metal Atox1 complex readily forms dimers that exhibit different (but still low energy) near-UV CD signals. Also for the Atox1 dimer complex is both Cys residues important as the Atox1 variant Cys15Ala, which bind Cu in parallel with dimer formation (using Cu as bridge interacting with one Cys from each protein), do not exhibit any unique CD features upon CisPt binding. Geometry optimization using DFT calculations demonstrate that CisPt interaction with Cys12 (the most exposed cysteine) is energetically favorable and upon optimization results in bond-formation with Cu. In analogy with our indications of a di-metal site, a recent study of Atox1 interactions with Au(I) (d^10^ configuration like Pt(II)) found that adducts containing one protein and two Au(I) ions were formed in large amounts [53].

Finally, we show that CisPt can be transferred between Atox1 and WD4 and that a heterodimer involving both proteins and CisPt is stabilized. This implies that CisPt binding to Atox1 *in vivo* may not be a dead-end species (ending up as an unfolded and aggregated species), but the metal may be transferred to a metal-binding domain of ATP7A/B (or other proteins, such as glutathione) before CisPt-triggered Atox1 unfolding kicks in. This may be the mechanism behind Cu-transfer protein mediated resistance to CisPt *in vivo*: despite low abundance of these proteins in the cells, by shuttling the drug further along the Cu-transfer pathway (notably ATP7A/B has capacity to bind up to six CisPt in its metal-binding domains), many Pt molecules may be taken care of by a limited number of Cu-transport proteins.

One may ask why Cu transport proteins play a key role in Pt detoxification as there are many other Cys-rich proteins and peptides in the cytoplasm. As a common thread in our experiments, we have noted that Pt binding to Atox1 is enhanced when Atox1 is in the holo-form, i.e., Cu-Atox1. Thus, if the presence of Cu helps attract the Pt ion to Atox1, this could be the explanation for the presence of a bias of Pt binding towards Cu transfer proteins in the cytoplasm. Future studies involving Pt transfer between Atox1 and WD domains *in the presence of Cu* are desired (work in progress).

## Supporting Information

Figure S1 Near-UV CD spectra for full titrations. Left column, CisPt titration to holo-protein. Right column, Cu titration to premixed protein-CisPt 1∶1. **A+B.** WT Atox1. **C+D.** Cys41Ala Atox1. **E+F.** 3Cys3Ala Atox1. **G+H.** Cys15Ala Atox1. **I+J.** Met10Ala Atox1.
(PDF)

Figure S2 SDS-gel analysis of apo-protein and holo-protein (1 eq. Cu pre-added to protein). **A.** WT Atox1. **B.** Cys41Ala Atox1. **C.** 3Cys3Ala Atox1. **D.** Cys15Ala Atox1. **E.** Met10Ala Atox1. **F.** WT WD4.
(PDF)

Figure S3 Analytical gelfiltration of WT Atox1. Black: 280 nm, Grey: 254 nm. **A.** Apo. **B.** Apo +1 eq. CisPt. **C.** Apo +5 eq. CisPt. **D.** (Apo +1 eq. CisPt) +1 eq. Cu. **E.** Holo. **F.** Holo +1 eq. CisPt. **G.** Holo +5 eq. CisPt.
(PDF)

Figure S4 Analytical gelfiltration of Cys41Ala Atox1. Black: 280 nm, Grey: 254 nm. **A.** Apo. **B.** Apo +1 eq. CisPt. **C.** Apo +5 eq. CisPt. **D.** (Apo +1 eq. CisPt) +1 eq. Cu. **E.** Holo. **F.** Holo +1 eq. CisPt. **G.** Holo +5 eq. CisPt.
(PDF)

Figure S5 Analytical gelfiltration of 3Cys3Ala Atox1. Black: 280 nm, Grey: 254 nm. **A.** Apo. **B.** Apo +5 eq. CisPt. **C.** Apo +1 eq. Cu. **D.** (Apo +1 eq. Cu) +5 eq. CisPt.
(PDF)

Figure S6 Analytical gelfiltration of Cys15Ala Atox1. Black: 280 nm, Grey: 254 nm. **A.** Apo. **B.** Apo +1 eq. CisPt. **C.** Apo +5 eq. CisPt. **D.** (Apo +1 eq. CisPt) +1 eq. Cu. **E.** Apo +1 eq. Cu. **F.** (Apo +1 eq. Cu) +1 eq. CisPt. **G.** (Apo +1 eq. Cu) +5 eq. CisPt.
(PDF)

Figure S7 Analytical gelfiltration of Met10Ala Atox1. Black: 280 nm, Grey: 254 nm. **A.** Apo. **B.** Apo +5 eq. CisPt. **C.** (Apo +1 eq. CisPt) +1 eq. Cu. **D.** Holo. **E.** Holo +5 eq. CisPt.
(PDF)

Figure S8 Analytical gelfiltration of WT WD4. Black: 280 nm, Grey: 254 nm. **A.** Apo. **B.** Apo +1 eq. CisPt. **C.** Apo +5 eq. CisPt. **D.** (Apo +1 eq. CisPt) +1 eq. Cu. **E.** Holo. **F.** Holo +1 eq. CisPt. **G.** Holo +5 eq. CisPt.
(PDF)

Figure S9 Overlay of chromatograms from analytical gelfiltration. Black: Transfer setup, Atox1-CisPt (monomer elutes at 14.2 ml) mixed with 0.5 eq. WD4 (monomer elutes at 12.8 ml). Blue: WD4 alone incubated with CisPt, resulting in monomers (12.8 ml) and dimers (11.3 ml). Red: Atox1alone incubated with CisPt, resulting in monomers (14.2 ml) and dimers (12.2 ml). The dimer observed in the transfer experiment (12.0 ml) does not match the elution of either WD4 or Atox1 homodimers but is found in between the homodimers, in strong support of a WD4-Atox1 heterodimer linked by CisPt.
(PDF)

Figure S10 Structural differences between NMR structures used for geometry optimizations. Superposition using backbone atoms for amino acid in the proximity of the Cu-atom of NMR structures 1 (A, purple), 12 (B, green) and 24 (C, orange). The residues shown including hydrogens (not shown) were used in the geometry optimization calculations.
(TIF)

Figure S11 Starting geometries compared to geometry optimized complexes. Starting geometries (in thin line) and geometry optimized complexes (in stick) between CisPt and Atox1, with distance indication between Cu (brown) and Pt (light blue). a) NMR structure A with CisPt conformation 2 (A2). b) NMR structure A with CisPt conformation 3 (A3). d) NMR structure B with CisPt conformation 1 (B1). d) NMR structure C with CisPt conformation 4 (C4).
(TIF)

File S1 (PDF)
