# Supplementary figures and images for: Determinants for Simultaneous Binding of Copper and Platinum to Human Chaperone Atox1: Hitchhiking not Hijacking
Source: PLoS One. 2013 Jul 30;8(7):e70473. doi: 10.1371/journal.pone.0070473 (PMC3728025; doi:10.1371/journal.pone.0070473)

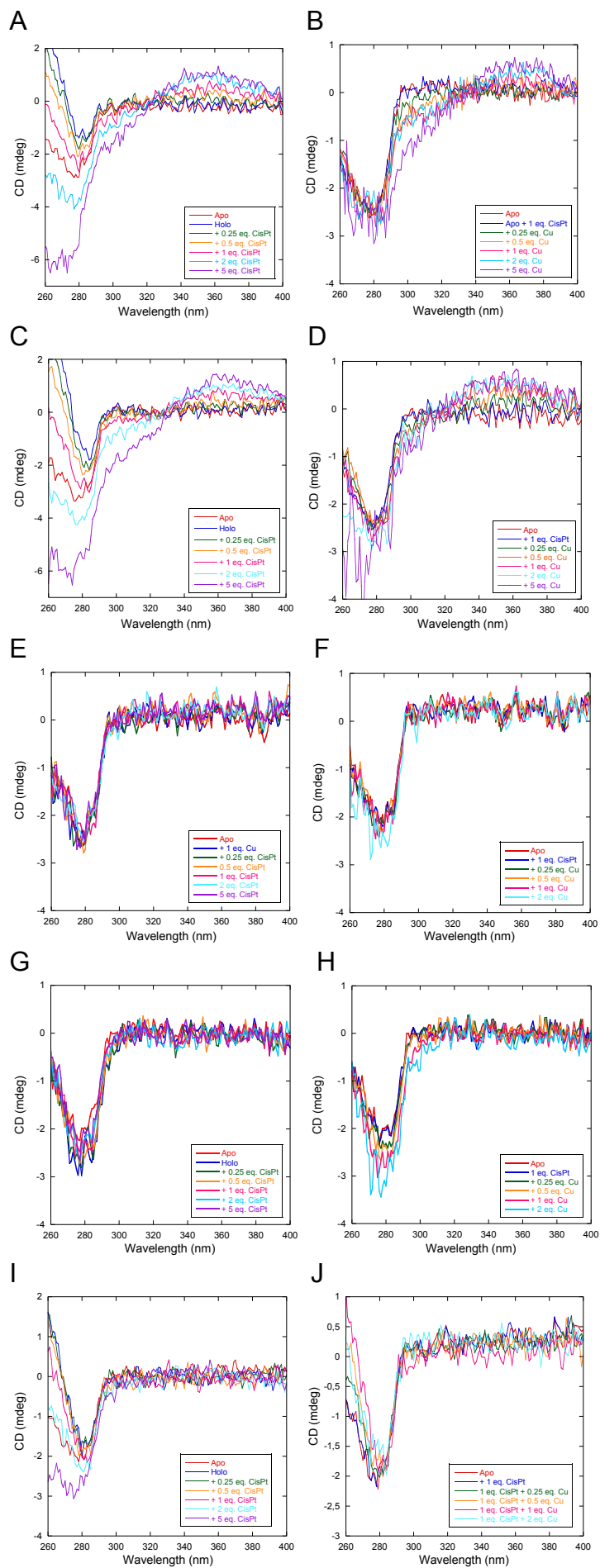

Supplement: Figure S1 — Left column, CisPt titration to holo-protein. Right column, Cu titration to premixed protein-CisPt 1∶1. A+B. WT Atox1. C+D. Cys41Ala Atox1. E+F. 3Cys3Ala Atox1. G+H. Cys15Ala Atox1. I+J. Met10Ala Atox1. (PDF) [file pone.0070473.s001.pdf]

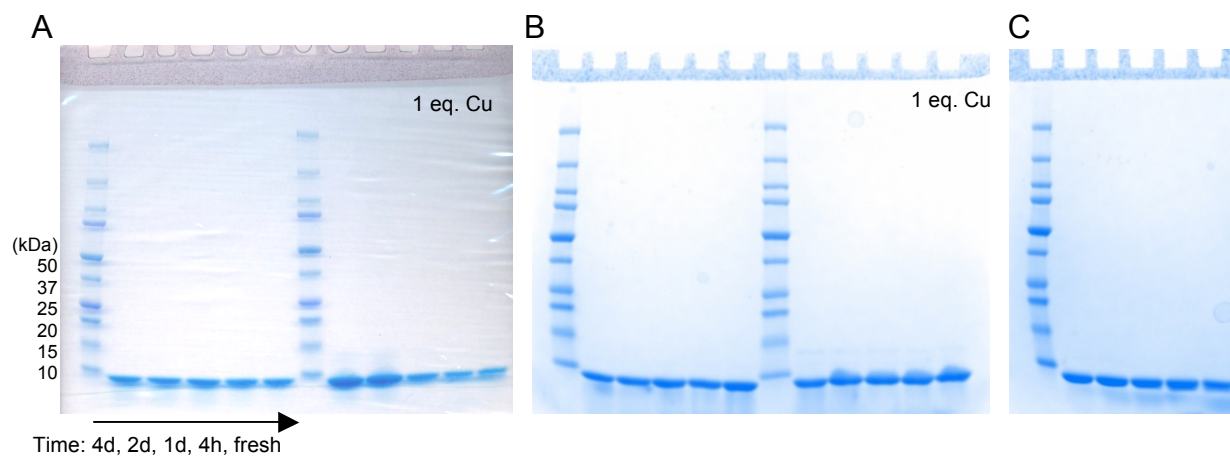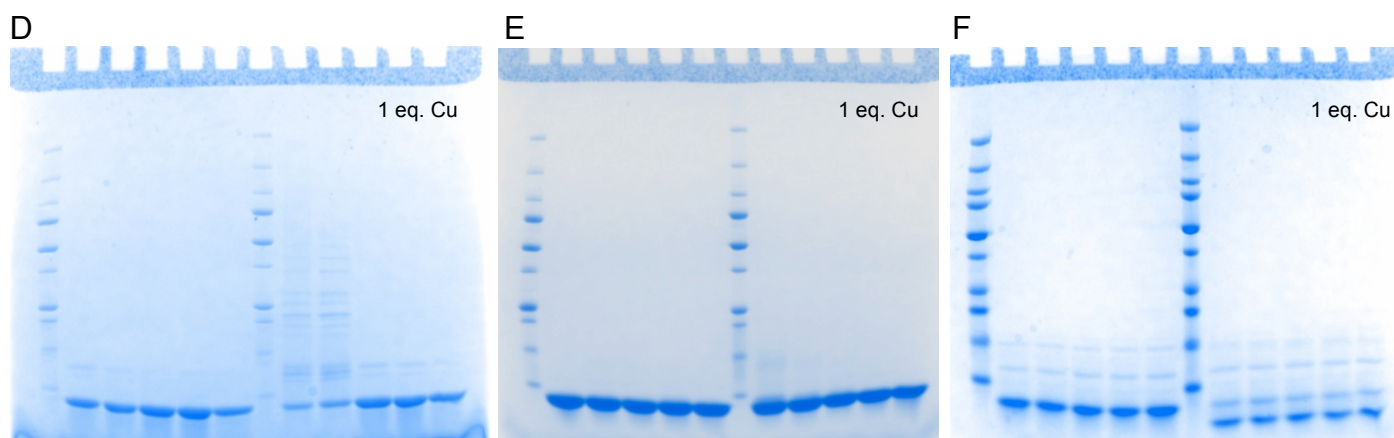

Supplement: Figure S2 — A. WT Atox1. B. Cys41Ala Atox1. C. 3Cys3Ala Atox1. D. Cys15Ala Atox1. E. Met10Ala Atox1. F. WT WD4. (PDF) [file pone.0070473.s002.pdf]

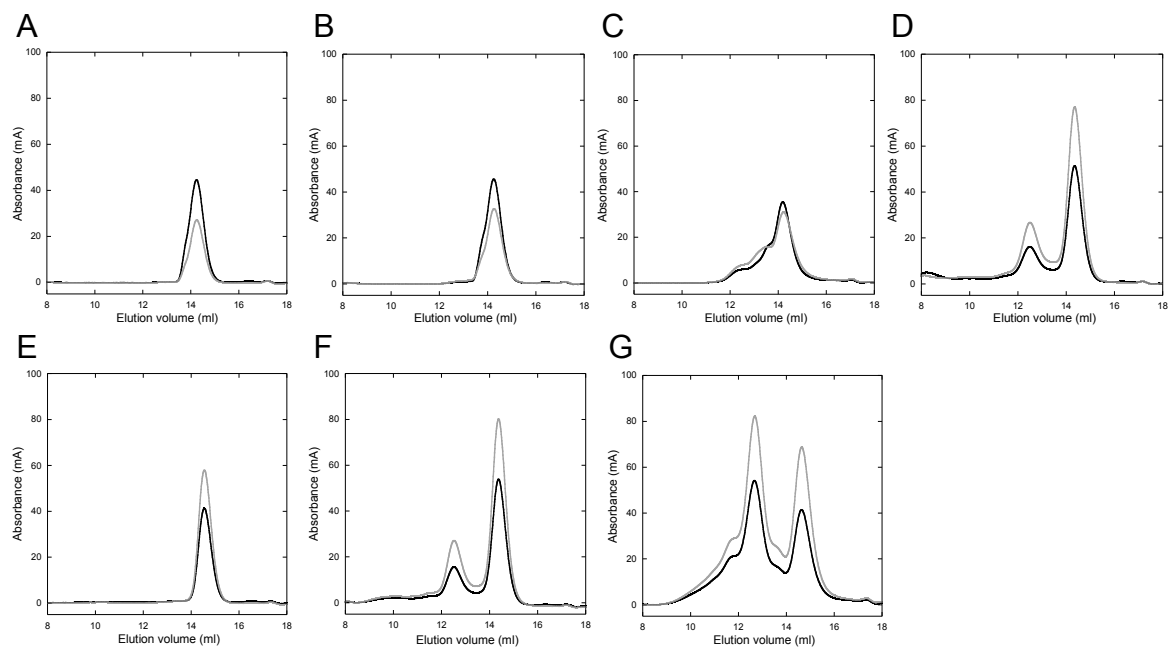

Supplement: Figure S3 — A. Apo. B. Apo +1 eq. CisPt. C. Apo +5 eq. CisPt. D. (Apo +1 eq. CisPt) +1 eq. Cu. E. Holo. F. Holo +1 eq. CisPt. G. Holo +5 eq. CisPt. (PDF) [file pone.0070473.s003.pdf]

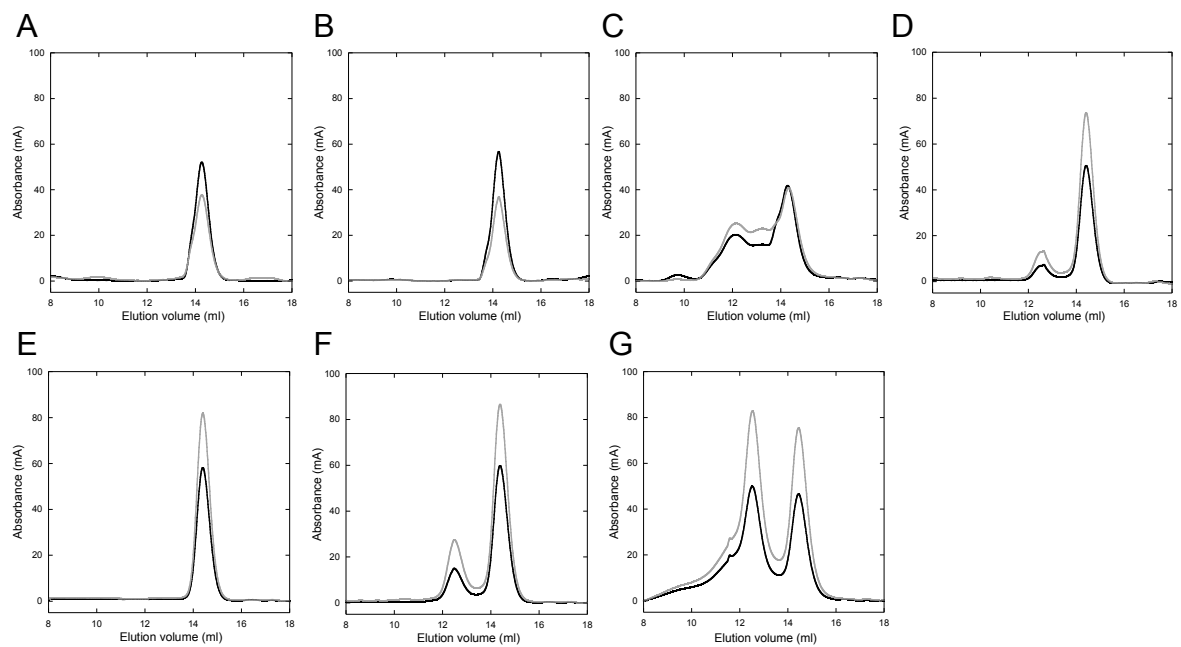

Supplement: Figure S4 — A. Apo. B. Apo +1 eq. CisPt. C. Apo +5 eq. CisPt. D. (Apo +1 eq. CisPt) +1 eq. Cu. E. Holo. F. Holo +1 eq. CisPt. G. Holo +5 eq. CisPt. (PDF) [file pone.0070473.s004.pdf]

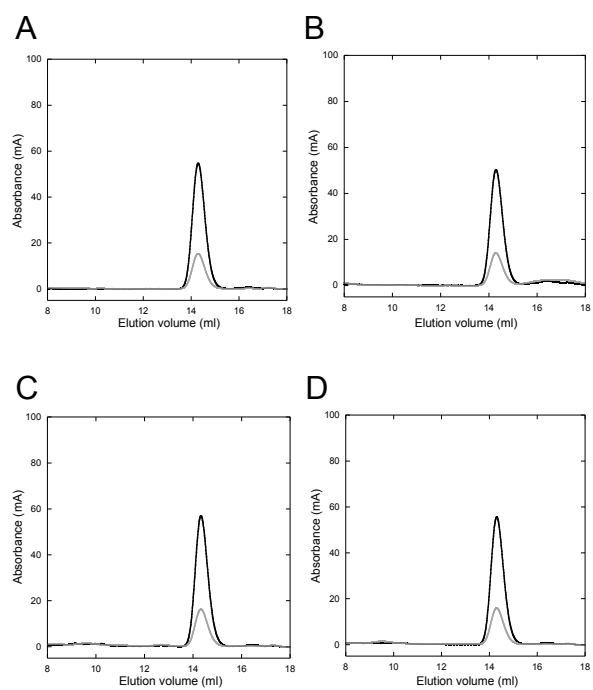

Supplement: Figure S5 — A. Apo. B. Apo +5 eq. CisPt. C. Apo +1 eq. Cu. D. (Apo +1 eq. Cu) +5 eq. CisPt. (PDF) [file pone.0070473.s005.pdf]

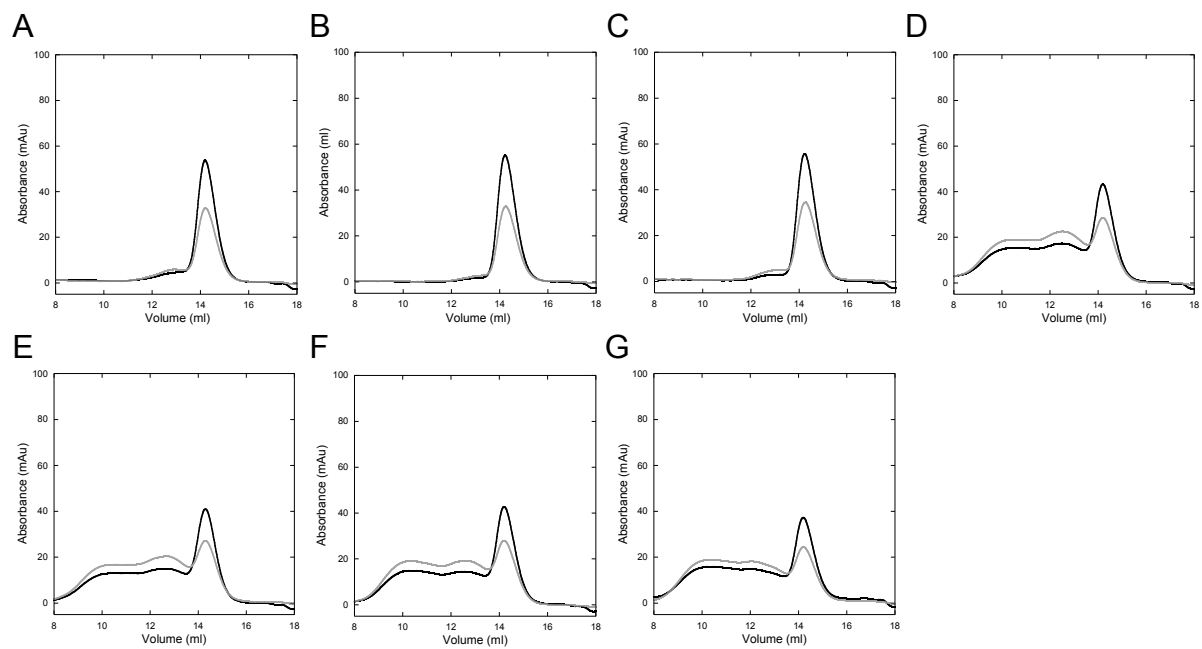

Supplement: Figure S6 — A. Apo. B. Apo +1 eq. CisPt. C. Apo +5 eq. CisPt. D. (Apo +1 eq. CisPt) +1 eq. Cu. E. Apo +1 eq. Cu. F. (Apo +1 eq. Cu) +1 eq. CisPt. G. (Apo +1 eq. Cu) +5 eq. CisPt. (PDF) [file pone.0070473.s006.pdf]

**A**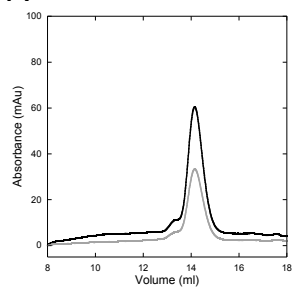**B**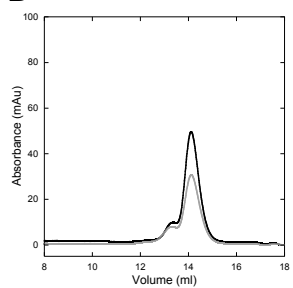**C**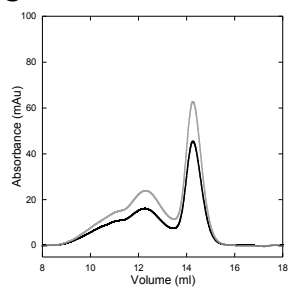**D**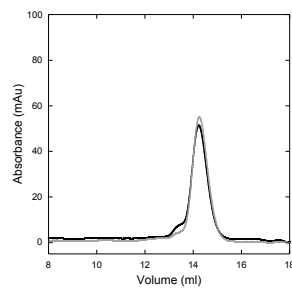**E**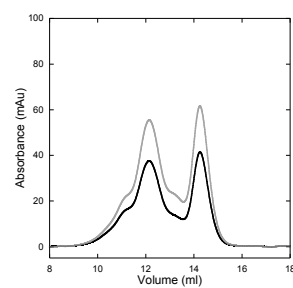

Supplement: Figure S7 — A. Apo. B. Apo +5 eq. CisPt. C. (Apo +1 eq. CisPt) +1 eq. Cu. D. Holo. E. Holo +5 eq. CisPt. (PDF) [file pone.0070473.s007.pdf]

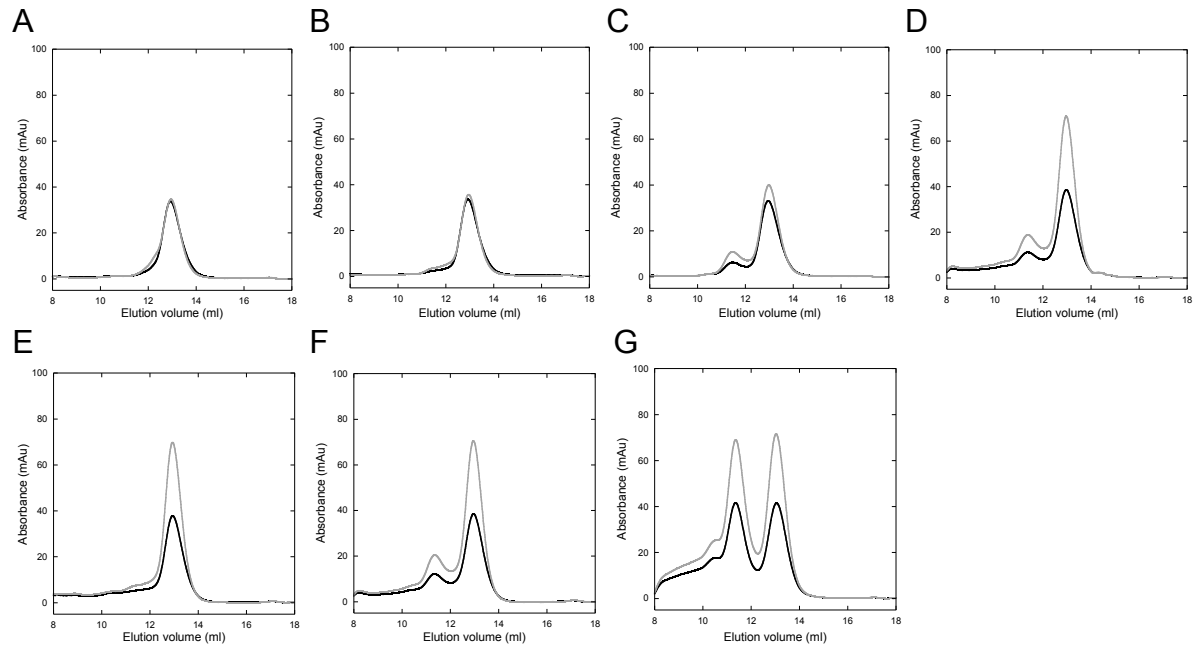

Supplement: Figure S8 — A. Apo. B. Apo +1 eq. CisPt. C. Apo +5 eq. CisPt. D. (Apo +1 eq. CisPt) +1 eq. Cu. E. Holo. F. Holo +1 eq. CisPt. G. Holo +5 eq. CisPt. (PDF) [file pone.0070473.s008.pdf]

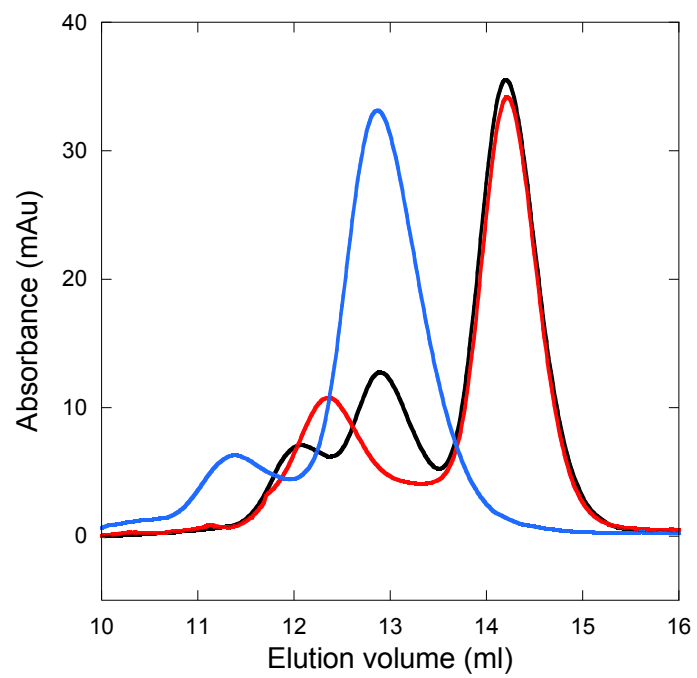

Supplement: Figure S9 — Black: Transfer setup, Atox1-CisPt (monomer elutes at 14.2 ml) mixed with 0.5 eq. WD4 (monomer elutes at 12.8 ml). Blue: WD4 alone incubated with CisPt, resulting in monomers (12.8 ml) and dimers (11.3 ml). Red: Atox1alone incubated with CisPt, resulting in monomers (14.2 ml) and dimers (12.2 ml). The dimer observed in the transfer experiment (12.0 ml) does not match the elution of either WD4 or Atox1 homodimers but is found in between the homodimers, in strong support of a WD4-Atox1 heterodimer linked by CisPt. (PDF) [file pone.0070473.s009.pdf]

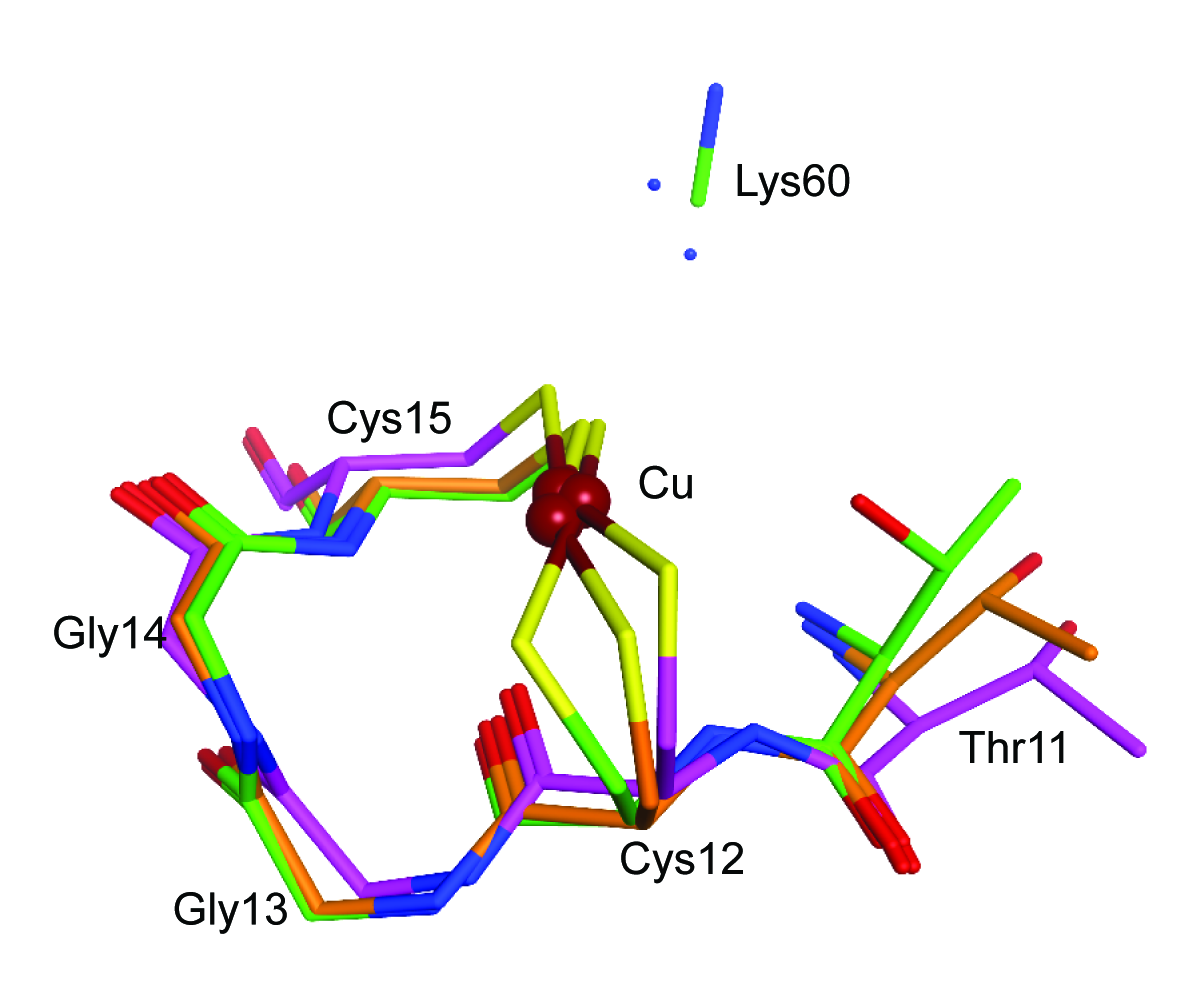

Supplement: Figure S10 — Superposition using backbone atoms for amino acid in the proximity of the Cu-atom of NMR structures 1 (A, purple), 12 (B, green) and 24 (C, orange). The residues shown including hydrogens (not shown) were used in the geometry optimization calculations. (TIF) [file pone.0070473.s010.tif]

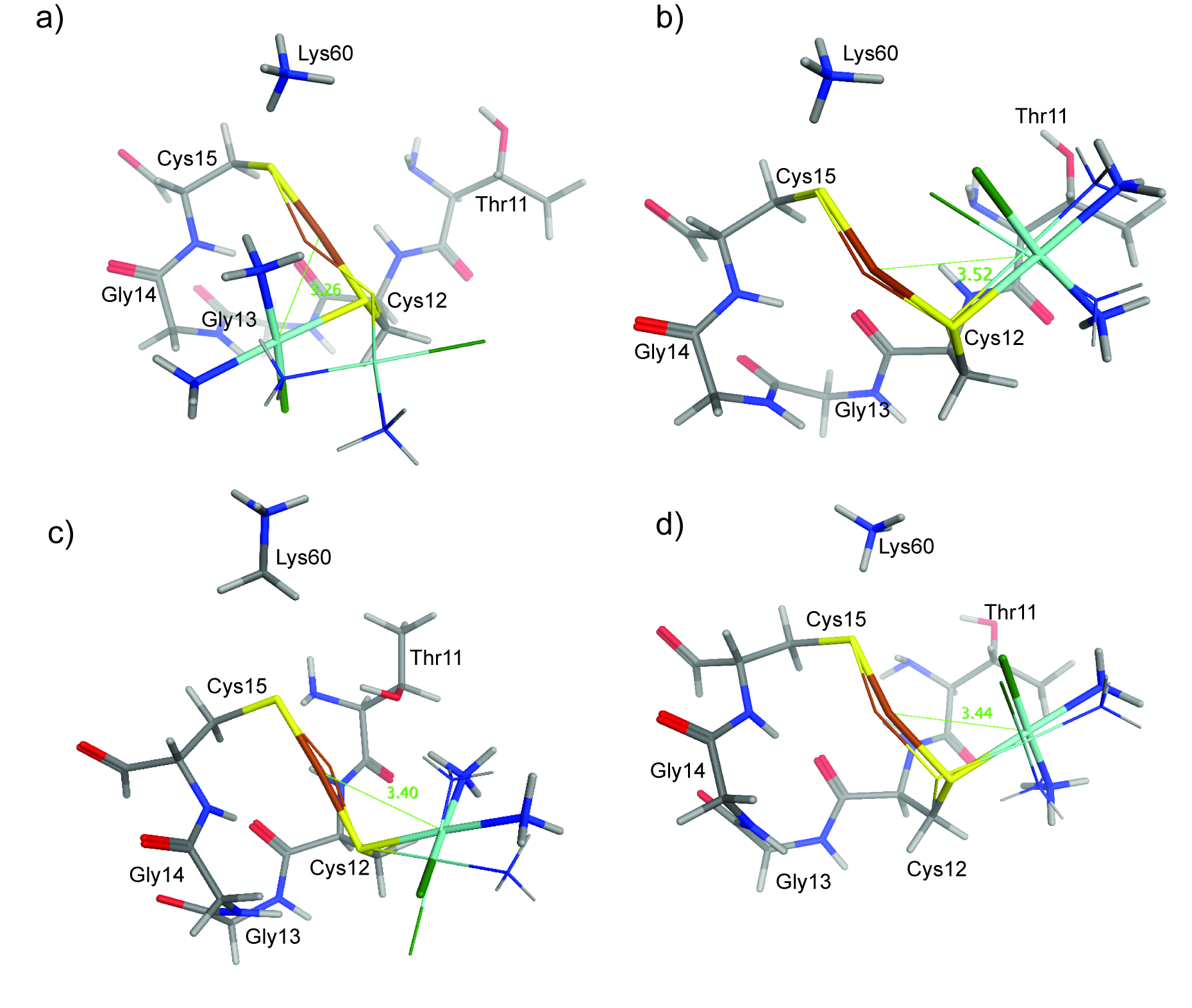

Supplement: Figure S11 — Starting geometries (in thin line) and geometry optimized complexes (in stick) between CisPt and Atox1, with distance indication between Cu (brown) and Pt (light blue). a) NMR structure A with CisPt conformation 2 (A2). b) NMR structure A with CisPt conformation 3 (A3). d) NMR structure B with CisPt conformation 1 (B1). d) NMR structure C with CisPt conformation 4 (C4). (TIF) [file pone.0070473.s011.tif]
