# Supplementary material for: Determinants for Simultaneous Binding of Copper and Platinum to Human Chaperone Atox1: Hitchhiking not Hijacking
Source: PLoS One. 2013 Jul 30;8(7):e70473. doi: 10.1371/journal.pone.0070473 (PMC3728025; doi:10.1371/journal.pone.0070473)
Supplement: File S1 — (PDF) [file pone.0070473.s012.pdf]

SUPPORTING INFORMATION

Determinants for simultaneous binding of copper and platinum to human chaperone Atox1: *hitchhiking not hijacking*

Maria E. Palm-Espling, C. David Andersson, Erik Björn, Anna Linusson and Pernilla Wittung-Stafshede

Contents

Method - Quantum mechanical calculations

Additional results including **Table S1 -** Quantum mechanical calculations

Quantum mechanical calculations - Method

Calculations were performed on the NMR structure of Atox1 (pdb code 1TL4 [1]) using three different structures (1 (A), 12 (B) and 24 (C)) that represented the structural diversity around the Cu^{I} binding-site present among the total of 30 NMR structures (**Figure S10**). $CisPt^{+}$ ($ClH_6N_2Pt^{+}$) was built into the structures assuming that the Cys12 sulfur had two available binding-sites (the third binds to Cu^{I}) where the Pt of $CisPt^{+}$ might bind. $CisPt^{+}$ was modelled in at these sites with a Pt-S distance of 2.25 Å and angles consistent with a tetrahedral sulphur geometry (i.e. angles of 109.5 °) and a square planar geometry for $CisPt^{+}$. For NMR structures A and C, both binding-sites of $CisPt^{+}$ to sulphur was modelled in two conformations differing by a rotation of 180 ° around the Pt-NH_3 axis causing an exchange of position for the chlorine and the opposing NH_3 group. Only one sulphur binding site was available in NMR structure B, the unavailable one was blocked by the protein. The protein structures were truncated to only include amino acids in the proximity of the Cu^{+} binding site including Thr11, Cys12, Gly13, Gly14, Cys15, and Lys60 (side chain N (protein structures A and C) or NCH_3 (protein structure B)). Protons were added at the truncation-positions and were minimized using Macro Model [2] with the OPLS-2005 force field.

The Atox1-$CisPt^{+}$ complexes were geometry optimized with Turbomole [3] in two steps using dispersion-corrected density functional theory (DFT-D3) [4] in gas phase with the b-lyp functional [5-7], a multiple grid size “m3”, basis set def2-SVP(Weigend 2005) (step1) and def2-TZVP(Weigend 2005) (step2), and an effective-core potential (ECP) def2-ecp for Pt and the Stuttgart-Koeln MCDHF RS [8] for Cu. $CisPt^{+}$, Cu and Cys12- and Cys15 sulphurs were allowed to move during optimization, all other atoms were fixated. Single point energies were calculated for each optimized structures of the Atox1-$CisPt^{+}$ complex, and Atox1 and $CisPt^{+}$ separately from the same complex, with DFT-D3 and the same settings as in geometry optimization step2. Interaction energies, ΔE, were computed by subtracting the summarized energy for $CisPt^{+}$ and Atox1 from the Atox1-$CisPt^{+}$ complex energy.

Quantum mechanical calculations - Results

Low-energy binding modes between $CisPt^+$ and Atox1 was computed by geometry optimization of Atox1-$CisPt^+$ complexes using DFT-D3 calculations. The initial binding modes (start geometry) were generated by placing the $CisPt^+$ at a probable binding location to the sulphur of Cys12, the only accessible binding partner of the two Cu-site cysteins. Three protein conformations (A, B and C, **Figure S10**) and several $CisPt^+$ starting geometries (A1-4, B1-2, and C1-4) used in the geometry optimizations. Interaction energies for all optimized complexes are displayed in **Table S1**. The complexes with the lowest ΔE were, in decreasing order, A3, A2, C4 and B1. These interaction energies are in good agreement with previously reported basis set superposition error-corrected interaction energies for the cis-$[Pt(NH_3)_2Cl(Cys\text{-}X)]^+$ complex of ~110 kcal/mol (460.24 kJ/mol) [9].

Table S1. Energies and Pt-Cu distances of geometry optimized Atox1-CisPt complexes, with the complexes with lowest ΔE in bold.

		Complex Energy (kJ/mol)	Protein Energy (kJ/mol)	$CisPt^+$ Energy (kJ/mol)	ΔE (kJ/mol)	Pt-Cu distance (Å)
NMR structure A	Conf. 1	-7915300	-6096744	-1818119	-437	3.467
	**Conf. 2**	**-7915320**	**-6096728**	**-1818118**	**-474**	**3.258**
	**Conf. 3**	**-7915338**	**-6096745**	**-1818111**	**-482**	**3.524**
	Conf. 4	-7915267	-6096744	-1818121	-402	3.728
NMR structure B	**Conf. 1**	**-8018472**	**-6199888**	**-1818124**	**-460**	**3.400**
	Conf. 2	-8018433	-6199888	-1818123	-422	3.518
NMR structure C	Conf. 1	-7915309	-6096740	-1818121	-448	3.598
	Conf. 2	-7915294	-6096760	-1818119	-416	3.436
	Conf. 3	-7915236	-6096706	-1818122	-408	3.697
	**Conf. 4**	**-7915327**	**-6096744**	**-1818122**	**-461**	**3.443**

The starting geometries and optimized structures A2 and A3 that had the lowest interaction energies (ΔE) between Atox1 and CisPt of all complexes are displayed in **Figure S11**. In these complexes, the average Pt-S distance was 2.37 Å, a distance slightly bigger than previously reported distances for the Pt-S bond in a CisPt-cystein complex of 2.32-2.35 Å [10]. The average Pt-Cu distance in these two complexes was and 3.39 Å and, in fact, the average Pt-Cu distance was 3.41 Å counting all low-energy ΔE complexes for all three protein structures A2, A3, B1 and C4 (**Figure S11**).

Interpreting the results from this computational study it is clear that there exists several Atox1-CisPt low energy complexes where the binding geometry of CisPt depends on the protein geometry. A general trend in the geometry optimizations was that the Cu atom moved closer to the electron rich carbonyl oxygen of Cys12, presumably because of the complementary electrostatic nature of the two atoms. As a consequence, Pt also moved to maintain an interaction with Cu. The distance between Pt and Cu in the optimized complexes may be small enough for the two atoms to form a metal-metal interaction. The metal-metal interaction distance reported here is longer that previously reported for d^8-d^{10} orbital overlap type Pt-Cu dative bonds (see main text). The use of a static protein structures may hinder the CisPt from achieving an optimal binding mode to Atox1 due to steric clashes between Pt ligands in CisPt and the protein residues.

References

1. Anastassopoulou I, Banci L, Bertini I, Cantini F, Katsari E, et al. (2004) Solution structure of the Apo and copper(I)-loaded human metallochaperone HAH1. Biochemistry 43: 13046-13053.
2. MacroModel v99225. Schrödinger, LLC. 120 West 45th Street, 17th Floor, Tower 45, New York, NY, 10036-4041.
3. TURBOMOLE v6.3.1, 2012. University of Karlsruhe and Forschungszentrum Karlsruhe GmbH, 1989-2007, TURBOMOLE GmbH, since 2007; available from www.turbomole.com.
4. Grimme S, Antony J, Ehrlich S, Krieg H (2010) A consistent and accurate ab initio parametrization of density functional dispersion correction (DFT-D) for the 94 elements H-Pu. J Chem Phys 132.
5. Ahlrichs R, Bär M, Häser M, Horn H, Kölmel C (1989) Electronic-structure calculations on workstation computers - the program system Turbomole. Chem Phys Lett 162: 165-169.
6. Schäfer A, Horn H, Ahlrichs R (1992) Fully optimized contracted gaussian-basis sets for atoms Li to Kr. J Chem Phys 97: 2571-2577.
7. Weigend F, Ahlrichs R (2005) Balanced basis sets of split valence, triple zeta valence and quadruple zeta valence quality for H to Rn: Design and assessment of accuracy. Phys Chem Chem Phys 7: 3297-3305.
8. Figgen D, Rauhut G, Dolg M, Stoll H (2005) Energy-consistent pseudopotentials for group 11 and 12 atoms: adjustment to multi-configuration Dirac-Hartree-Fock data. Chem Phys 311: 227-244.
9. Zimmermann T, Burda JV (2010) Reactions of cisplatin with cysteine and methionine at constant pH; a computational study. Dalt Transac 39: 1295-1301.
10. Zimmermann T, Chval Z, Burda JV (2009) Cisplatin Interaction with cysteine and methionine in aqueous solution: Computational DFT/PCM study. J Phys Chem B 113: 3139-3150.
